# A Diet Profiling Algorithm (DPA) to Rank Diet Quality Suitable to Implement in Digital Tools—A Test Study in a Cohort of Lactating Women

**DOI:** 10.3390/nu15061337

**Published:** 2023-03-09

**Authors:** Marta Alonso-Bernáldez, Andreu Palou-March, Rocío Zamanillo-Campos, Andreu Palou, Mariona Palou, Francisca Serra

**Affiliations:** 1 Alimentómica S.L. Camí de na Pontons. s/n (Pol.11, Parc 3), 07310 Campanet, Spain; 2 Laboratory of Molecular Biology, Nutrition and Biotechnology (Nutrigenomics, Biomarkers and Risk Evaluation Group), University of the Balearic Islands, 07121 Palma, Spain; 3 Health Research Institute of the Balearic Islands (IdISBa), 07120 Palma, Spain; 4 CIBER of Physiopathology of Obesity and Nutrition (CIBEROBN), Carlos III Health Institute (ISCIII), 28029 Madrid, Spain; 5 Primary Care Research Unit of Mallorca, Balearic Islands Health Service, Carrer de l’Escola Graduada 3, 07002 Palma, Spain

**Keywords:** diet quality, diet profiling, dietary advice, diet algorithm, eHealth, breast milk leptin

## Abstract

Although nutrient profiling systems can empower consumers towards healthier food choices, there is still a need to assess diet quality to obtain an overall perspective. The purpose of this study was to develop a diet profiling algorithm (DPA) to evaluate nutritional diet quality, which gives a final score from 1 to 3 with an associated color (green-yellow-orange). It ranks the total carbohydrate/total fiber ratio, and energy from saturated fats and sodium as potentially negative inputs, while fiber and protein are assumed as positive items. Then, the total fat/total carbohydrate ratio is calculated to evaluate the macronutrient distribution, as well as a food group analysis. To test the DPA performance, diets of a lactating women cohort were analyzed, and a correlation analysis between DPA and breast milk leptin levels was performed. Diets classified as low quality showed a higher intake of negative inputs, along with higher energy and fat intakes. This was reflected in body mass index (BMI) and food groups, indicating that women with the worst scores tended to choose tastier and less satiating foods. In conclusion, the DPA was developed and tested in a sample population. This tool can be easily implemented in digital nutrition platforms, contributing to real-time dietary follow-up of patients and progress monitoring, leading to further dietary adjustment.

## 1. Introduction

Unhealthy dietary patterns based on energy-dense and/or low-nutrient foods are one of the most modifiable risk factors directly associated with morbidity and mortality of noncommunicable diseases (NCD). Recent data show that 11 million deaths worldwide were attributable to dietary risk factors in 2017 alone [1]. Furthermore, the overall obesity prevalence has increased over the years reaching 41.5%, a trend that is expected to keep rising [2]. This staggering figure prompts an urgent need to identify new strategies aiming to change and improve dietary habits worldwide.

Concerning global approaches, different ways to assess nutritional profiling are being developed to identify the healthfulness of foods and to help consumers make more informed food choices when grocery shopping [3,4]. The Ofcom model developed by the UK Food Standards Agency [5] was one of the first approaches in this regard, leading the way in the development of new applications, such as food labeling. This application of nutrition profiling is an effective strategy to reduce the intake of unhealthy nutrients [6]. In particular, it has been shown that front-of-package (FoP) food labels can help consumers from all ages make healthier choices [7,8,9] and are already being implemented in different types and forms across the world [10,11,12]. Nutri-Score is a FoP labeling system, developed and implemented by the French government [13], made up of colors and letters that make the classification of pre-packed products very visual and easy to understand in terms of healthfulness [14]. This strategy has been compared with other nutrient-profiling systems and validated by different studies, showing its effectiveness in terms of improving diet quality [4,10,15,16,17,18] and increasing unprocessed foods’ purchases [19]. However, the Nutri-Score algorithm is still an open concept and its refinement has been suggested to better align its outcome with dietary guidelines [20,21,22]. Overall, it is clear that nutrient-profiling systems can help consumers to make healthier choices, but the ranking of individual food products does not guarantee adherence to a healthy and balanced diet. Therefore, an approach focused on ranking the overall diet rather than individual products would be more suitable and comprehensive for this purpose [23].

Thus, the aim of this study was to develop a dietary profiling system/tool by defining an algorithm able to assess the nutritional quality of the diet and suitable for implementation in nutrition-related apps. This would allow us to take advantage of the fast and easily interpretable requirements of digital media, without losing the accuracy of more classical nutritional approaches, while giving support in a user-friendly manner.

## 2. Materials and Methods

### 2.1. Development of the Diet Profiling Algorithm

**Figure 1:**
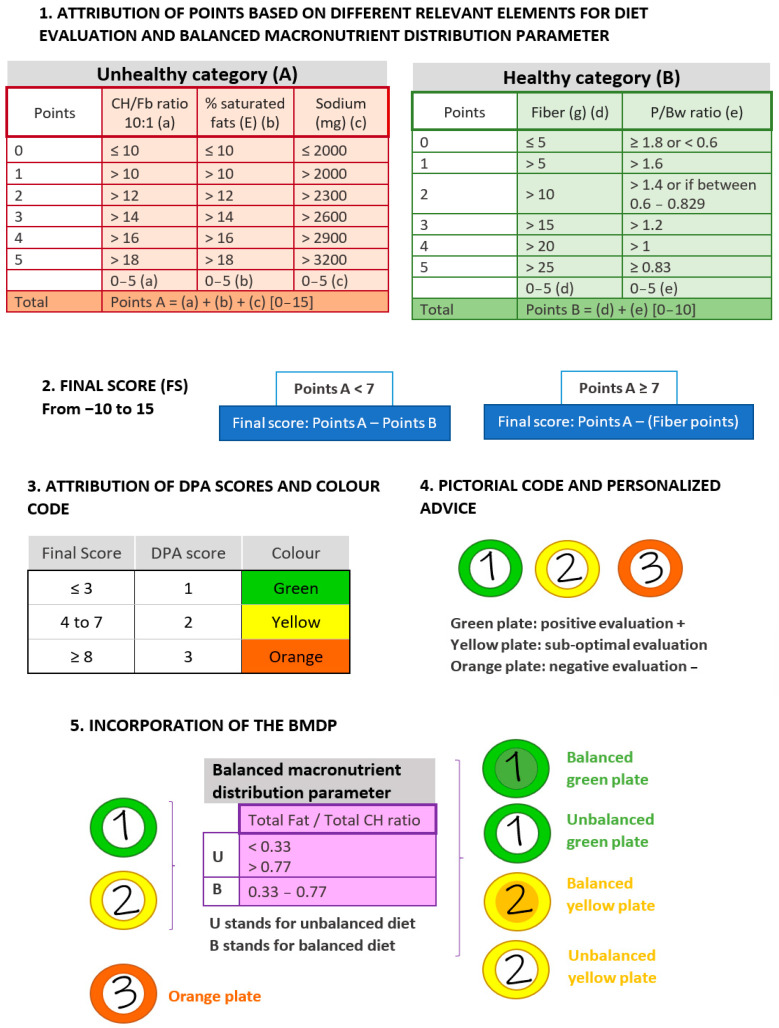
Components and scores of the DPA, a diet profiling algorithm to assess diet quality. The figure shows the sequential checkpoints considered by the DPA, displaying the points attributed and the range of application and the proposed final outputs. Abbreviations: DPA: diet profiling algorithm; CH: carbohydrate; Fb: fiber; E: energy; P/Bw: protein/body weight ratio; BMDP: balanced macronutrient distribution parameter.

Development of the diet profiling algorithm (DPA) was initially based on the main concepts and structure of the Nutri-Score [13]. In order to adapt this profiling system from individual food products to overall diets, the modification of specific parameters of the algorithm were implemented. A score from 0 to 5 was assigned to each nutrient, ratio, or parameter considered, and the cut-offs for score assignation were mainly based on the European Food Safety Authority (EFSA) and World Health Organization (WHO) guidelines [24,25]. Two categories of nutrients were defined based on the accumulated evidence of their impact on health, which are described in the following sections. In addition, an estimation of balanced macronutrient distribution was introduced (see Section 2.1.4), as well as certain guides aiming to go into deeper personalized advice (Figure 1).

#### 2.1.1. Unhealthy Nutrients Assessment

Total carbohydrate/total fiber ratio (CH/Fb), saturated fatty acids (SFA, % energy), and sodium (mg) were the three factors considered in this category as their excess intake may lead to unhealthy events [3] (Figure 1, Point 1A). Firstly, the CH/Fb ratio can help to determine how much of the total dietary intake of carbohydrates may be accompanied by a healthy food matrix, contributing to a better assessment of nutrient density [20,26]. The CH/Fb ratio gives a good proxy to distinguish between free or added sugars (e.g., from sugary beverages) and sugars naturally present in foods (e.g., from fruit). Intakes equal to or greater than 1 g of fiber per 10 g of carbohydrate can be considered as a healthy ratio (10:1) [27,28]. In the DPA, CH/Fb ratio cut-offs ranged from ≤10 to >18 (the best and worst score in this category, respectively) and was ranked from 0 to 5 points.

Concerning fat, we focused on the % of total energy coming from SFA. Since the EFSA recommends its intake to be as low as possible [24], and in accordance with the WHO [25], the threshold of 10% was selected as the most adequate. Consequently, cut-offs ranged from ≤10 to >18% and were ranked as described above from 0 to 5 points.

To evaluate influence of sodium intake, the EFSA recommendation of ≤2000 mg of sodium per day for adults was adopted [29]. Thus, the best score was attributed to ≤2000 mg of sodium intake, whereas >3200 mg of sodium was assigned to the highest score. See Figure 1 for the intermediate range values.

#### 2.1.2. Healthy Nutrients Assessment

In this category, protein and fiber were considered as beneficial nutrients to rate the DPA (Figure 1, Point 1B). To assess protein intake, the intake ratio of protein/body weight (P/Bw) was used. The EFSA has established 0.83 g/Kg bw/day of protein for adults as the population reference intake (PRI) that will meet the requirements of 97.5% of the individuals [30]. Hence, a ratio of 0.83 was established as the best and ranked with a 5 (the best score in this category). Higher ratios were ranked with lower scores, with >1.8 as the highest cut-off. In addition, to contemplate protein deficit, the average intake (AI) set by the EFSA [30] was used as a specific cut-off. Then, daily intakes <0.6 g/Kg bw were qualified with 0, whereas intermediate intakes (0.6–0.829) were ranked with 2.

Fiber intake was considered following the EFSA guidelines of ≥25 g of total fiber as a daily adequate amount for the correct function of the organism [31]. Therefore, values of >25 g/day of fiber obtained the highest score and values of ≤5 g/day the lowest.

#### 2.1.3. Attribution of DPA Scores and Color Code

Points obtained from the assessment of the three unhealthy parameters were added (A value) and the same was done within the healthy category (B value) (Figure 1, Point 2). If the unhealthy points are lower than 7, the final score (FS) of the diet is Points A minus Points B. On the contrary, a score of ≥7 in Points A may be indicative of a high presence of unhealthy nutrients; thus, protein will not count as a positive item to avoid bonification for unhealthy food sources. In this case, the FS will result in only Points A—fiber points. This FS is then converted into a DPA score, which goes from 1 to 3 (from best to worst diet quality) (Figure 1, Point 3). If the FS ≤ 3: DPA = 1; from 4 to 7: DPA = 2; and if FS ≥ 8: DPA = 3. Each DPA score is attributed either to green, yellow, or orange, respectively.

The final representation of diet assessment is intended to be integrated into a digital platform and, for example, to be able to be displayed to the user in a very intuitive manner within an app. A proposal would be coding the DPA score with graphic symbols, making use of pictorial codes, for instance, such as a colored plate. Plates of intuitive colors would be able to efficiently represent DPA scores (Figure 1, Point 4).

#### 2.1.4. Balanced Macronutrient Distribution Parameter (BMDP)

Aiming to introduce a parameter to evaluate the distribution of macronutrients in the diet, the ratio of total fat and total carbohydrate (F/CH) was defined, referred to the percentage of total energy provided by each of these nutrients (Figure 1, Point 5). The EFSA has established 20–35% and 45–60% of total energy intake coming from fats and carbohydrates, respectively, as part of adequate daily intakes [24]. Thus, an F/CH ratio between 0.33 and 0.77 was classified as suitable. These cut-offs would correspond to what an acceptable macronutrient distribution in a high-carbohydrate or high-fat diet would be, respectively. Diets with an F/CH ratio of <0.33 or >0.77 were classified as “U” (meaning unbalanced), while diets with an F/CH ratio between 0.33 and 0.77 were classified as “B” (standing for balanced). The information obtained from the BMDP is used as an additional informative factor, promoting more personalized advice. This can be implemented in the graphical coding, emphasizing the attribution of colors for instance.

### 2.2. Food Group DPA Assessment

Dietary data from food groups allow for the analysis of food group consumption and its comparison with a reference. The software DIAL v3.0 (Alce Ingeniería, Madrid, Spain) was used for this purpose. The software intrinsically divides food into the food groups stipulated by the EFSA [3] plus three more, resulting in: cereals; fats and oils; milk and dairy products; sugars, sweets and pastries; beverages; meat and meat products; fruits and nuts; eggs; fish; legumes; vegetables; and miscellaneous. Beverages included every drink different from water and milk. Miscellaneous included agglutinated precooked products, appetizers, sauces, and dietetic products. To establish a food group pattern as reference, meals contained in three daily menus proposed by the Mediterranean Diet Foundation [32] were selected and analyzed with DIAL. The contribution of each food group to total energy intake was obtained for every menu and their average was set as the reference amount of total energy coming from the above-cited food groups. As expected, the three menus were ranked as green plates by the DPA.

**Figure 2:**
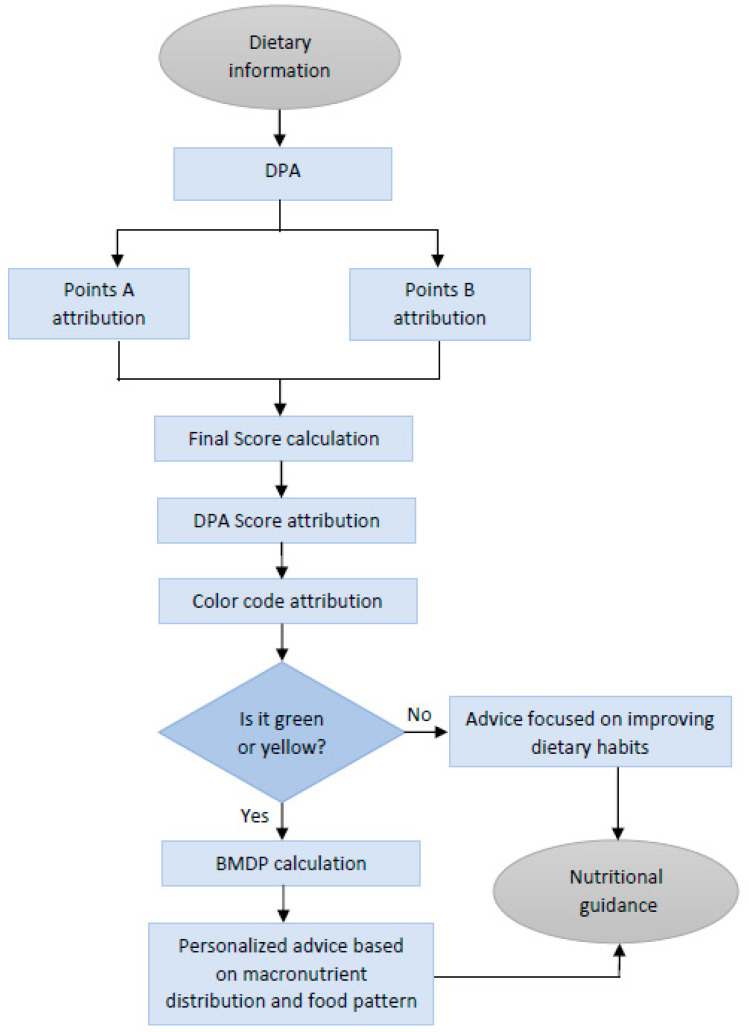
Flow chart of the DPA, a diet profiling algorithm to assess diet quality. Nutritional information is processed by the algorithm, taking into consideration the constraints fixed. Dietary records are used as input data to ultimately result in nutritional guidance at different levels of personalization. Abbreviations: DPA (diet profiling algorithm); BMDP: balanced macronutrient distribution parameter.

In summary, the DPA uses dietary information as the input to start the assessment, quantifies and qualifies the diet, and then reaches a final verdict whether the diet can be considered healthy or not. This is used to generate the nutritional guidance for each specific user. In case of an affirmative answer, the DPA proceeds to calculate the BMDP and analyze the food groups to assess whether an optimal diet is being followed or whether there is room for improvement. In any case, personalized advice adjusted to the consumer wishes and preferences can be delivered. If the answer is negative, then the DPA proceeds to give advice focused on improving the dietary habits. Thus, feeding the DPA with dietary data (see below) allows the running of the algorithm and its use as a tool for nutritional guidance and education. whether is in nutritional consultation or in a nutrition-related app (Figure 2).

### 2.3. DPA Implementation and Related Output

**Figure 3:**
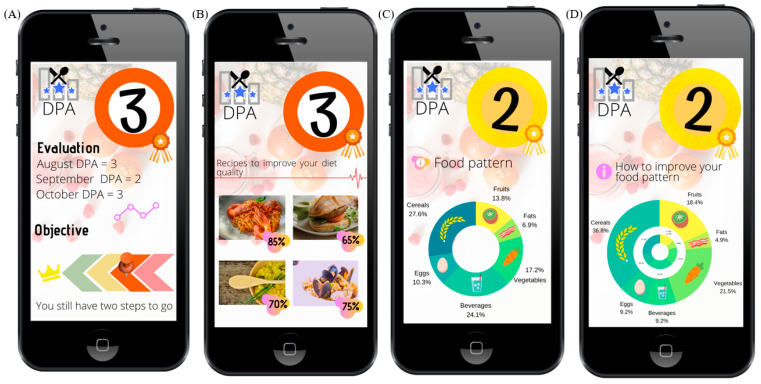
A sample of a mobile interface screens showing the DPA implementation as colored plates. (**A**) DPA score and the progress of the user; (**B**) tailored recipe suggestions; (**C**) the actual food groups; (**D**) some recommended improvements in order to provide personalized advice.

The DPA has been designed to be used on digital platforms that record users’ food intake on a daily basis (i.e., with food pictures and post-image recognition technology or other methods). The information on recipes, dishes, and respective amounts would be then converted to nutritional data by the software of that specific platform. Then, this would go into the DPA to perform the diet assessment, give appropriate dietary advice, and suggest more appropriate menus/dishes (Figure 3).

To facilitate follow-up by average users, three output levels are proposed. The orange (DPA = 3) diet would be characterized by very poor diet quality, generally due to prioritizing unhealthy foods rather than nutrient-rich foods. Since diet quality would be so low, the balanced macronutrient distribution parameter would not be calculated, nor the food groups. The immediate advice would be to improve dietary habits by highlighting the parameters that are contributing to this bad score (e.g., explaining to the user the weak aspects of their current diet (i.e., excess salt, low fiber, etc.). Then, high-quality menus representative of healthy diet(s) would be suggested by the app, encouraging diet changes by focusing on good food elections aiming to upgrade the DPA score (Figure 3A,B).

In the case of a yellow (DPA = 2) score, the quality of the reported diet would be sub-optimal, and some specific aspects could be improved. Then, to give more personalized advice, two other parameters would be analyzed: the BMDP and the food groups. Hence, as a second step, the BMDP would be calculated and incorporated in the graphical display. In case of a “U”, an alert to watch macronutrient distribution would be made and specific advice would focus on modifying the current diet to a better fit, in contrast with the more drastic change that arises from DPA = 3. As a third step, food group analysis is performed and graphically displayed. The output would focus on recipes/tips involving food groups to be recommended by the app to better suit the food group pattern of reference (Figure 3C,D).

If a green (DPA = 1) classification is obtained, the quality of the diet would be close to optimal. Then, BMDP, as well as the food groups, would be calculated. Ideal users would get a “B” BMDP and fitted food groups. No major changes to their diet would be necessary. Therefore, the app would propose healthy menus similar to the ones they eat and like, and it would follow-up any deviations from this set point. If macronutrients are correctly distributed (indicated by obtaining a “B” in the BMDP assessment), but the food groups do not fit with the set reference, then the advice would be to prioritize recipes containing a more balanced food group distribution, for example, by keeping macronutrient distribution while promoting higher legume intake as opposed to meat products, for instance.

However, if a green score is obtained with unsatisfactory macronutrient distribution (“U” in the BMDP), a change in the dietary pattern would be promoted towards balancing the macronutrient distribution at the same time as the food groups fit the reference.

### 2.4. Testing the Performance of the DPA

#### 2.4.1. Population Characteristics and Diet Evaluation

The DPA has been designed to empower consumers to make healthier choices, so it could be tested in any group of the general population. In the present study, the DPA performance was initially tested using previously collected data from a cohort of lactating women, which was part of an obesity-related research in our lab. The recorded diets of 59 lactating women were submitted to screening. The cohort was recruited within the observational Nutrigen-11 study carried out between 2011 and 2014 in three health centers in Mallorca (Spain) (agreement approval IB 1645/11). Adult women without any infectious illness and wishing to participate were considered for inclusion in this study. Women were recruited after delivery when they attended the midwife consultation. Then, personal interviews were scheduled at months 1, 2, and 3 of lactation in which anthropometric measurements and the diet were recorded. In addition, a breast milk sample was collected at these time points when possible. Concerning dietary intake, three 24 h dietary recalls (24 h), one per month, were recorded on paper and transferred to the computer. Energy, nutrient composition, and food groups were obtained by using the aforementioned dietary software DIAL. This software applies Atwater factors to estimate energy intake (9 kcal/g for fat, 4 kcal/g for protein and carbohydrate, 2 kcal/g for fiber, and 7 kcal/g for alcohol). The mean values of this nutritional information were introduced as input to the DPA to obtain the diet score of each participant. To contemplate the 19 g extra protein requirement stipulated by the EFSA recommendations in the first months of lactation [24], the protein intake of each woman was adjusted by subtracting this quantity from the total protein amount consumed. The resulting grams were used for the DPA score’s calculation as if it were a normal adult population.

#### 2.4.2. Determination of Leptin in Milk as Biomarker

Breast milk leptin is an interesting biomarker to study the influence of the maternal diet on milk composition as it influences metabolic imprinting, childhood development, and future health status [33]. Leptin concentration was determined as previously described [34].

#### 2.4.3. Statistical Analysis

The SPSS v21 for Windows (SPSS, Chicago, IL, USA) software was used for data analysis. To assess differences between DPA ranked scores, one-way analysis of variance (one-way ANOVA) followed by the least significance difference (LSD) post hoc test was used. If homogeneity of variances was violated, variables were logarithmically transformed. Single comparisons between DPA scores were assessed by Student’s *t*-test. Moreover, to determine the association between breast milk leptin and body mass index (BMI) for the different DPA scores, Spearman’s rank correlation measures were performed. Data are presented as the mean ± standard error of the mean (±SEM). The threshold of significance was set at *p*-value ≤ 0.05.

## 3. Results

### 3.1. Diet Characterization and DPA Scores of the Population

**Figure 4:**
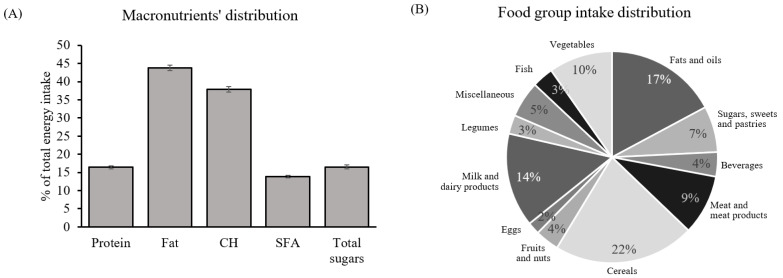
General dietary characterization of the cohort: (**A**) macronutrient distribution. Percentage of total energy intake coming from protein, fat, CH, SFA, and total sugars; (**B**) food groups. Percentage of total energy coming from twelve different food groups. Data are mean ± SEM (*n* = 59). Abbreviations: SFA: saturated fatty acids; CH: carbohydrates.

The mean (±SEM) population age was 32 ± 0.45 years old, with an average BMI of 24.1 ± 0.58 kg/m^2^. However, 36% of the cohort was overweight with a BMI equal to or greater than 25 kg/m^2^. Daily energy intake was 2152 ± 70 kcal, which nearly met what the EFSA dietary guidelines recommend for lactating women aged between 18 and 39 [24]. However, the population did not show a balanced macronutrient distribution (Figure 4A) regardless of BMI. Carbohydrate intake accounted for 37.9% of total daily energy (far from the 45–60% recommended), which included 16.5% of energy coming from sugars (the sum of digestible sugars present in all the food groups analyzed), while total fat intake surpassed the recommended 20–35%, reaching 43.8% with a relevant proportion of SFA (13.8%). Meanwhile, protein intake was 16.4% (almost meeting the actual guidelines, 10–15%) [24]. Regarding food groups, the majority of energy intake came from cereals, with 22% of total energy intake, followed by 17% of fats and oils, 14% of milk and dairy products, and minor contributions from the other food groups (Figure 4B).

**Figure 5:**
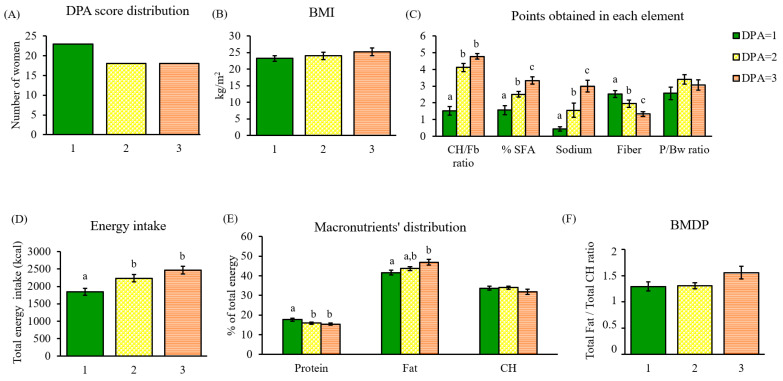
Dietary characterization of the cohort through DPA. (**A**) DPA score distribution; (**B**) BMI of women classified by their DPA score; (**C**) points obtained in each DPA element and classified by DPA score; (**D**) total energy intake (kcal) associated with the DPA score; (**E**) percentage of total energy coming from macronutrients in each DPA score; (**F**) total fat/total carbohydrate (F/CH) ratio defining the BMDP in each DPA score. Data are mean ± SEM (*n* = 23, 18, and 18, respectively). Statistics: groups with different letters are significantly different (LSD post hoc one-way ANOVA, *p*-value ≤ 0.05). Abbreviations: DPA: diet profiling algorithm; BMI: body mass index; CH: carbohydrate; Fb: fiber; SFA: saturated fatty acids; P/Bw: protein/body weight; BMDP: balanced macronutrient distribution parameter.

The DPA scores of the cohort showed that 39% of the population were following diets that could be considered of good nutritional quality and, therefore, classified as green plates. The rest of the cohort was equally ranked as 31% yellow and 31% orange (Figure 5A). A tendency to increased BMI as diet quality decreased was observed, although it did not attain statistical significance (Figure 5B).

### 3.2. Nutrient and Macronutrient Distribution Assessment through DPA

To characterize the algorithm performance, healthy and unhealthy parameters were individually analyzed. Total points obtained were significantly different between DPA scores in the case of the CH/Fb ratio (*p* < 0.001), % SFA (*p* < 0.001), sodium (*p* < 0.001), and fiber (*p* < 0.001), whereas the P/Bw ratio did not present significant differences between DPA scores (Figure 5C).

Therefore, the diets of women ranked with a green score (DPA = 1) were characterized by the lowest intake of negative nutrients (CH/Fb ratio, % SFA, and sodium) and the highest fiber intake in comparison with the rest of the DPA scores. Yellow and orange scores (DPA = 2 and 3) showed greater intake of negative nutrients, specifically of simple carbohydrates, tripling the CH/Fb ratio punctuation of green plates. Overall, orange plates (DPA = 3) ate more nutrients categorized as negative and less fiber than those obtaining lower DPA scores.

Regarding total energy intake, poor nutritional quality positively correlated with energy (*p* < 0.001) (Figure 5D). Although more energy does not necessarily mean unhealthy, the algorithm detects that this population substantially exceeded the recommended fat intake as mentioned above (Figure 4A), which increased with DPA scores (*p* = 0.011) (Figure 5E). On the contrary, protein intake decreased with poorer diet quality (*p* = 0.032) (Figure 5E).

The BMDP, aiming to assess macronutrient balance, was estimated in the population and, according to the results, none of the screened diets were balanced, even when obtaining the best DPA score. The lowest scores (DPA = 1 and 2) had the same F/CH ratio (≈1.30), whereas DPA = 3 had a higher ratio (1.56), far from the stipulated suitable range (Figure 5F).

### 3.3. Food Group Assessment through DPA

**Figure 6:**
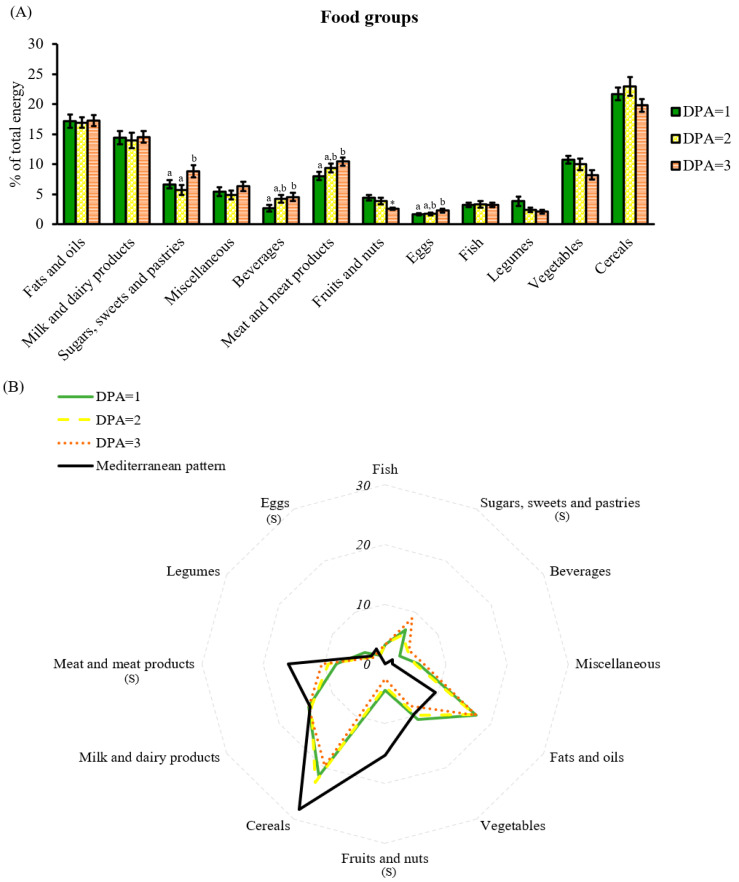
Food group analysis by DPA score. (**A**) Percentage of total energy coming from food groups classified by DPA score; (**B**) comparison of the reference food groups based on the Mediterranean diet with the food groups shown in the cohort and classified by DPA score. Data are mean ± SEM DPA scores 1, 2, and 3 (*n* = 23, 18, and 18, respectively). Statistics: (**A**) groups with different letters are significantly different (LSD post hoc one-way ANOVA, *p*-value ≤ 0.05); if not significant, single comparisons between DPA = 1 and DPA = 2 or 3 were assessed by Student’s *t*-test (*, *p*-value ≤ 0.05); (**B**) S, differences between DPA scores and Mediterranean diet (one-way ANOVA, *p*-value ≤ 0.05). Abbreviations: DPA: diet profiling algorithm.

Concerning food groups, DPA = 3 was associated with the worst food groups, attaining the highest energy intake from the unhealthier ones, such as sweets, beverages, and meat products. Whereas, the most recommended to maintain good health, such as vegetables, fruits and nuts, and legumes were displaced (Figure 6A). This pattern was in contrast with the preference observed with the food groups in diets ranked as green (DPA = 1) and yellow (DPA = 2). Accordingly, poor diet quality ranked by DPA was also reflected in food groups; particularly, women classified under the best score tended to choose less palatable, but more satiating foods, and rich in fiber and micronutrients. Remarkably, the DPA recognizes that the diets followed by the cohort are far from the Mediterranean dietary pattern; no diet perfectly suited the Mediterranean food groups, as a relative deficit in cereals, meat, and fruits and nuts was shown, whereas an excess in fats and oils, sugars, sweets and pastries, and beverages was present (Figure 6B).

### 3.4. Breast Milk Leptin Assessment

**Figure 7:**
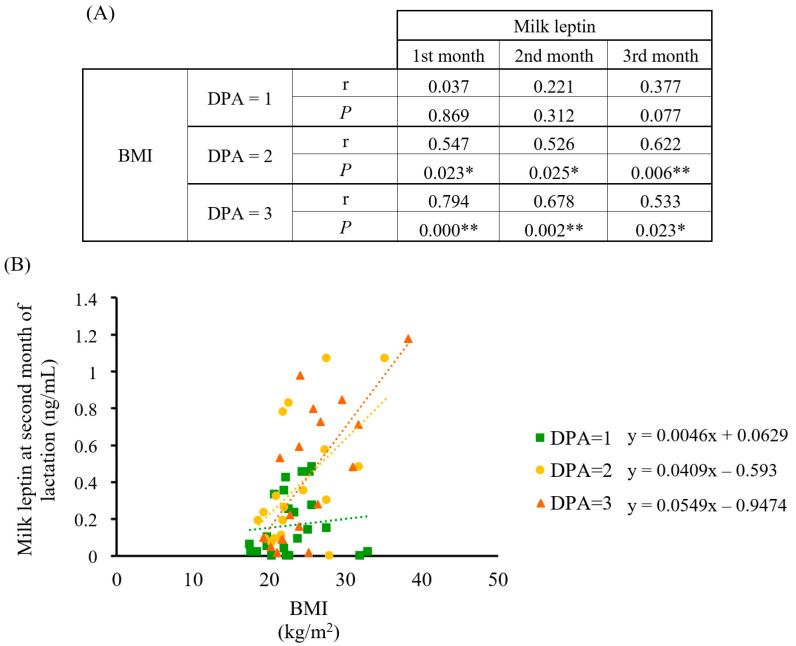
Correlation analysis between BMI and breast milk leptin concentration in the cohort classified by DPA score (*n* = 23, 18, and 18, respectively). (**A**) Spearman’s correlation data between BMI and leptin at the first, second, and third month of lactation by DPA score; (**B**) scatter plot and linear regression trend lines with data from the second month of lactation. Statistics: Spearman’s correlation test, * *p*-value ≤ 0.05 (** = *p*-value < 0.01). Abbreviations: BMI: body mass index; DPA: diet profiling algorithm; r: Spearman’s rank correlation coefficient; *p*: *p*-value.

Previous results from our group have underlined the relevance of adequate leptin levels in breast milk for infant development [35,36,37,38]. Therefore, we tested the involvement of maternal diet quality on milk leptin and analyzed its relationship with the DPA score. Results showed that diet quality influenced the association between maternal BMI and breast milk leptin levels (Figure 7). Specifically, a positive and statistically significant correlation appeared in women whose diets were ranked as DPA = 2 and 3 and their BMI, in contrast with the lack of correlation shown between the best diet quality (DPA = 1) and BMI. Therefore, a high-quality maternal diet appeared to counteract BMI’s influence on milk leptin concentration throughout lactation, whereas a higher BMI would imply higher and sustained milk leptin levels under inadequate diets.

## 4. Discussion

Obesity affects millions of people globally and has become a major public health issue. Recent studies have associated the increasing rates of overweight people and people with obesity with unhealthy dietary patterns. These are mainly characterized by low vegetables, fruits, and whole grain intake [1,39,40], in addition to high ultra-processed food and drink (UPFD) content [41,42,43]. Different approaches have been adopted by international organizations and health-related institutions [44] to empower consumers to choose healthier food items, such as nutritional labeling in pre-packed foods. Moreover, several diet quality indicators (DQIs) and dietary recording methods have been developed to evaluate diet quality. The recently updated Healthy Eating Index [45] and the Mediterranean Diet Score [46] are good examples based on a number of representative items. DQIs have been successfully implemented in population studies and have enabled the development of guidelines to improve the health and nutritional status in such populations. However, the individual’s DQI outcome cannot be considered intuitive or comprehensible for average citizens if they have access to it [47]. In fact, data on food consumption indicate that people have little knowledge about nutrition and have difficulties in following a balanced diet.

Nutrition in the digital age can use modern technologies to perform heavy computational load, analyze diet composition and quality, and reach individual users in a more personalized manner. In the last decade, digital technology developments have enabled the launching of uncountable nutrition-related mobile apps offering new features, which facilitate dietary recording and diet assessment in comparison with traditional methodologies [48,49,50,51]. These new systems may allow self-monitoring and also constant feedback from and to the user, constituting a dynamic exchange of information very valuable to dietitians to succeed in long-term dietary behavior changes [49,52,53]. However, in order to give personalized nutritional advice on the ubiquitous digital platforms, new, easy, and intuitive diet assessment methods are needed. In this context, a dietary profiling algorithm (DPA) has been developed to implement dietary intake analysis in digital nutrition tools and platforms. The aim is to empower people to eat healthier and motivate them to improve dietary and lifestyle habits in a customized way. Development of the DPA fits the current demand for nutritional advice in mobile devices along with real-time and user-friendly diet assessment tools. The DPA permits a quick and easily interpretable outcome of nutritional status at a glance. Diets are ranked in 3 DPA scores (from 1 to 3), each one with a different color attribution, which can be represented with pictorial elements, such as colored plates (DPA = 1, green; DPA = 2, yellow; DPA = 3, orange), enabling a first visual impression of diet quality and guiding dietary guidelines and recipes for improvement.

To assess the performance and utility of the DPA, diets of a cohort of lactating women were analyzed by the algorithm. Although more than half of the screened population presented a good or moderate diet quality (DPA = 1 and 2, respectively), the DPA pointed out that 31% of the cohort had poor diet quality (DPA = 3). The DPA efficiently highlighted the deficiencies of the diet, as the score positively correlated with the points attributed by the algorithm to unhealthy nutrients, and negatively with fiber. This indicates that the DPA was able to reflect the fact that low quality diets can be characterized by a large amount of free sugars, saturated fat, and sodium, and less fiber. In fact, the BMDP showed that diets classified by the DPA with the worst nutritional quality were significantly associated with higher fat intake at the expense of carbohydrate and protein intake.

The introduction of a second level of analysis using the concept of the BMDP revealed that no women followed a balanced diet regardless of DPA score. Indeed, food group analysis showed that no diet perfectly suited the Mediterranean pattern. Suboptimal consumption of cereals, meats, fruits, and nuts was observed, along with the excessive consumption of fats and oils, sugary foods, and beverages. Therefore, although general recommendations on the pinpointed nutrients were met in the lower DPA scores, the food choices driving them may not be in accordance with the best diet quality, which gives room for dietary improvement. However, it is important to recall that the perfect combination of food groups does not exist, and many different food groups may be perfectly healthy. Nonetheless, fruits and nuts, vegetables, and whole grains, are considered the fundamental pillars in every diet and, therefore, should be present in any healthy and desirable food pattern [54].

Next, our interest was focused on milk leptin and its relationship with the dietary profile to confirm the utility of the DPA and to obtain further insight on the impact of diet quality during the perinatal period. In this regard, leptin has been widely used as an early predictive marker for obesity and metabolic syndrome since its involvement in early programing [33]. Breast milk leptin positively correlates with leptin serum in lactating mothers [55], which, in turn, positively correlates with the mother’s BMI [55,56,57] and body fat [58]. Therefore, obese women could be providing inadequate milk leptin levels to their infants [34,56], and this is of relevance since leptin has been associated with obesity prevention at the early stages of life through milk supply [38,59,60,61]. The DPA score was tested as a tool to relate maternal diet quality to milk leptin levels. Interestingly, the fact that women’s BMI with diets ranked as green plates correlated with milk leptin in a weaker manner, especially if overweight or obese, gives support to the protective effect of the best diet quality by maintaining adequate leptin concentrations. In contrast, DPA scores >1 did not show this beneficial effect. These results highlight the importance of maternal diet quality during lactation, since providing optimal leptin concentration to newborns is critical for their correct development and metabolic programing [33].

In this work, a diet profiling algorithm, called DPA, has been developed and its potential usefulness as a tool to assess diet quality was tested in a population. At this developmental step, one limitation of the algorithm is that it does not consider other nutrients or compounds that may be relevant for health status, such as polyunsaturated fatty acids [62]. However, the DPA considers nutrients whose information is usually regarded in nutritional databases or food labels, which makes it easy to implement. Still, with the current technology, macro- and micronutrient profiles can be easily obtained from databases, and thus, the intake of other essential nutrients (such as vitamins or minerals) could be assessed and introduced in future versions. Nutrient cut-offs were established based on the population reference intake (PRI) and general recommendations for adults. Nonetheless, these cut-offs can be adapted for populations with different requirements or recommendations, such as protein in weightlifters, for instance. Moreover, the Mediterranean food pattern was selected as a reference model, targeting the specific population tested. However, other food patterns, such as vegetarian, can be considered as adequate in a healthy diet and easily implemented in a diet profiling algorithm such as the DPA. Furthermore, it would be interesting to test the DPA in the general population to confirm its performance. Concerning this last point, the DPA score has been implemented into the Mefood platform (https://www.mefood.io, accessed on 15 January 2023), a precision nutrition software to guide health professionals, which introduces more personalized recommendations for their clients according to their specific characteristics. In this setting, the score has been automatically implemented thanks to the access to the food composition database of the recipes/menus suggested by the platform with good preliminary results.

## 5. Conclusions

A diet profiling algorithm (DPA) has been developed as a good DQI in the present work to rank overall diets, and ideally designed for digital nutrition platforms in order to provide a visual and intuitive outcome of diet quality. The DPA can be automatically implemented by combining the use of new technologies coupled with the appropriate food database (e.g., by nationality) in order to extract the nutrient information to mathematically process the DPA. Then, a nutrition-related app can integrate the diet quality assessment and show the colored plates to the user in a friendly and individualized manner, reinforcing the good habits and promoting improvements where necessary. This tool may contribute to the empowerment of consumers concerning healthy eating habits, better product choices, and diets.

## Data Availability

The data presented in this study are available on reasonable request from the corresponding author. The data are not publicly available due to the consent provided by participants on the use of confidential data.
